# Investigación formativa y producción científica del docente odontológico peruano

**DOI:** 10.21142/2523-2754-1002-2022-109

**Published:** 2022-06-27

**Authors:** Ivo Luna-Mazzola, Rosmery Lorena Estela-Zamora, Manuel Antonio Mattos-Vela

**Affiliations:** 1 Sociedad Científica de Estudiantes de Odontología, Facultad de Odontología, Universidad Nacional Mayor de San Marcos. Lima, Perú. ivo00lunamazzola@gmail.com, rlorena.e.z@gmail.com Universidad Nacional Mayor de San Marcos Sociedad Científica de Estudiantes de Odontología Facultad de Odontología Universidad Nacional Mayor de San Marcos Lima Peru ivo00lunamazzola@gmail.com rlorena.e.z@gmail.com; 2 Facultad de Odontología, Universidad Nacional Mayor de San Marcos. Lima, Perú. mmattosv@unmsm.edu.pe Universidad Nacional Mayor de San Marcos Facultad de Odontología Universidad Nacional Mayor de San Marcos Lima Peru mmattosv@unmsm.edu.pe

**Keywords:** docentes de odontología, facultades de odontología, investigación, actividades científicas y tecnológicas, comunicación y divulgación científica, faculty dental, schools dental, Research, scientific and technical activities, scientific communication and diffusion

## Abstract

La investigación formativa y la producción científica docente son formas muy tempranas de enseñar el proceso investigativo, el cual implica una cultura de investigación que se debe instaurar desde el pregrado, de acuerdo con su fin, que es la difusión del conocimiento generado mediante una publicación científica. Para ello, se deben superar las principales trabas que la producción científica docente presenta. En las universidades peruanas que imparten la carrera de Odontología, la investigación formativa ha sido impulsada para fortalecer la futura investigación en el ámbito de las ciencias de la salud, y la producción científica docente resulta de suma importancia para el avance de la ciencia y la implicación del alumnado con dicha causa. El objetivo del presente trabajo es realizar una revisión sobre algunos aspectos de la producción científica docente y la investigación formativa en las facultades de odontología peruanas.

## Abstract (en)

Formative research together with scientific teaching production are very early ways of teaching the research process, which implies the research culture that must be established from the undergraduate level, taking into account its purpose, which is the dissemination of knowledge generated through scientific publication, for which it must seek to overcome the main obstacles that faculty scientific production presents. In the Peruvian universities that teach dentistry, formative research has been promoted to strengthen future research in the field of health sciences, being the faculty scientific production of utmost importance for the contribution to the advancement of science and implication of the students with that cause. The aim of this paper is to carry out a review on some aspects of faculty scientific production and formative research in Peruvian dental schools.

## INTRODUCCIÓN

La ciencia puede tener un carácter básico -busca el entendimiento de nuestro entorno mediante la generación de nuevos conocimientos- o aplicado -busca utilizar el conocimiento obtenido de las ciencias básicas para la generación de tecnologías o procedimientos-, pero es en la integración de ambos que se puede obtener un progreso social, como se da en el caso de las ciencias de la salud ^1^. 

La producción científica es también entendida como la materialización del conocimiento generado, con lo que cumple el objetivo de la investigación científica; esto permite conocer y solucionar los problemas científicos. Dicha producción es más que un conjunto de documentos almacenados, pues se le considera como la finalidad de las actividades académicas y científicas ^2^.

La investigación formativa es una función sustantiva de la enseñanza superior, es decir, todo universitario que egresa debe haber desarrollado habilidades investigativas durante su formación. Debido a esto, en diversas universidades se optado introducir en el pregrado cursos que integran conocimientos relacionados con la investigación formativa como objeto de estudio y como un camino de solución para los problemas de la profesión ^3^. En el contexto de la salud bucal, las facultades de odontología están reforzando sus procesos investigativos gracias a la formación de profesionales con grados de maestría y doctorado en ciencias básicas y salud pública, la divulgación de resultados en eventos científicos y un aumento considerable en la cantidad de publicaciones en revistas de alto impacto ^4^. 

Sin embargo, y teniendo en consideración la producción científica universitaria tanto docente como estudiantil -ya que estas se complementan-, el Perú ha reportado niveles bajos de investigación en ciencias de la salud ^5^^-^^6^, ya es superada en más del doble por España, que actualmente lidera la producción científica dentro de los países de habla hispana ^7^. Específicamente, en el campo de la odontología, los resultados no se pueden considerar distantes o mejores: Aquino Canchari ^8^ encontró que el 32,5% de los decanos de facultades y directores de escuelas de odontología peruanas habían publicado solo un artículo en sus vidas. Castro *et al*. ^9^ hallaron que la producción científica estudiantil en el pregrado odontológico era baja, lo que refleja una débil influencia docente en este ámbito.

Por tanto, el presente artículo tiene como objetivo realizar una revisión sobre algunos aspectos de la producción científica docente y la investigación formativa en las facultades de odontología peruanas.

## MATERIALES Y MÉTODOS

Se realizó una búsqueda bibliográfica sobre la investigación formativa y la producción científica docente en facultades de odontología peruanas. Las bases de datos consultadas fueron Science Direct, Medline y SciELO, así como los buscadores Scopus, PubMed y Google Académico. Se recolectó información de los últimos 10 años (entre enero de 2011 y diciembre de 2021) empleando las siguientes palabras de búsqueda: “Docentes de Odontología”, “Facultades de Odontología”, “Investigación” y “Producción científica”. Los términos en inglés fueron: “Faculty, Dental”, “Schools, Dental”, “Research” y “Scientific production”. Al conjugar los términos, se tuvo cuidado de mantener los siguientes: “Docentes de odontología” e '' investigación”, para delimitar bien la información obtenida. 

Tras la exploración de la literatura, la selección se inició con el análisis de títulos y resúmenes. Para ello, se tuvo en cuenta que dichas fuentes de información cubrieran alguno de los siguientes aspectos: importancia de la producción científica universitaria, rol del docente investigador, producción científica del docente odontológico peruano, trabas para la producción científica, investigación formativa y científica, importancia de la investigación formativa e investigación formativa en las facultades de odontología peruanas. También se consideró que los artículos aborden la relación entre la producción científica del docente universitario y la investigación formativa en el pregrado. Posteriormente, se realizó la lectura de los documentos con el fin de seleccionarlos: se incluyeron 27 fuentes entre artículos de revisión, artículos originales, tesis de grado y editoriales publicados tanto en español como en inglés. Los artículos de otro tipo o idioma fueron descartados para la presente revisión narrativa. 

## RESULTADOS Y DISCUSIÓN

### Importancia de la producción científica del docente universitario

Uno de los roles de la universidad es el aplicativo, que se produce, en parte, a través de la producción científica. Dentro de esta, tanto estudiantes como docentes cumplen funciones importantes desde los primeros años. Los docentes motivan a los estudiantes en el ámbito investigativo y constituyen un ejemplo al ser autores de publicaciones científicas en revistas de alto impacto. Sin embargo, se hace evidente la necesidad de una mejoría en este sistema al ver reportes en la literatura en los que hasta un 50% de los estudiantes de instituciones de educación superior consideran que sus conocimientos sobre redacción de artículos científicos son regulares y que más de la mitad de ellos opina lo mismo respecto de sus conocimientos sobre búsqueda de información ^9^. 

Además, se debe considerar que la producción científica docente favorece el desarrollo de la institución en la que se realiza. Pese a esto, la literatura muestra que se requiere impulsar la investigación en los líderes universitarios ^8^. 

No se debe dejar de mencionar que, en parte, una constante producción científica realizada en centros universitarios podría llevar a una posición ventajosa frente a otras instituciones afines al ámbito investigativo, lo que permitiría una mayor adquisición de proyectos de investigación, propuestas de colaboración interinstitucional, financiamiento y otros, como resultado del prestigio que conlleva una producción científica constante y de calidad.

### Rol del docente investigador en la producción científica universitaria

El rol docente ha evolucionado desde la mera transmisión del conocimiento hacia la facilitación del aprendizaje. Es así que el término “mediador” hace justicia al papel que se busca que los docentes cumplan hoy en día. El trasfondo de este cambio yace en la actual realidad de que la información se encuentra disponible y accesible para casi cualquier usuario, además de que esta se actualiza constantemente, lo que expande aún más la brecha de lo que una persona puede abarcar y, por tanto, transmitir/enseñar, y a lo que realmente se puede acceder para llegar a conocer. En dicho contexto, la investigación no se detiene, por lo que la gran cantidad de conocimientos que genera imposibilita mantenerse actualizado en todas las áreas. Por tanto, los docentes, como mediadores del conocimiento, deben introducir a sus estudiantes en el campo que enseñan y, a fin de complementarlo y profundizarlo, incentivar la lectura de artículos científicos referentes a estos temas.

Al ser un guía para el aprendizaje, el docente debe encontrarse preparado en conocimiento, práctica y experiencia de lo que va a enseñar. Además, la importancia de su actuar aumenta en tanto se les considera como modelos para aquellos que llenan sus aulas. Ser docente universitario implica contribuir al propósito de la universidad; por tanto, el rol investigativo queda inmerso dentro de sus funciones y, en sentido inverso, la investigación contribuye a la docencia, ya que permite llevar las experiencias obtenidas en dicho ámbito al salón de clases, complemento de la docencia con la investigación. 

Morales ^10^ indica que, respecto de los nuevos programas educativos mundiales, que priorizan la investigación como medio para la resolución de las problemáticas sociales en determinado ámbito, las escuelas odontológicas se encuentran en un proceso de adaptación y, por consiguiente, los docentes que imparten clases en ellas adquieren un rol protagónico. Por tanto, dicho autor propone una trilogía de órdenes para el docente universitario: preparación de profesionales competentes para el mercado laboral, formación de estudiantes que busquen la verdad mediante la investigación y que sean capaces de proyectar el saber en el escenario social. De aquí se desprende un factor clave para la producción científica universitaria: el binomio docente-investigador.

Además, gran parte de la importancia de que el docente sea investigador radica en cómo esto podría impulsar a sus estudiantes hacia el ámbito investigativo. Ello debido a que, principalmente en el pregrado, son los docentes con quienes los estudiantes tienen su más temprana aproximación hacia la investigación ^11^. De esta manera, el mérito está en “fomentar el pensamiento crítico y creativo en el estudiante para el planteamiento de problemas y búsqueda de soluciones enmarcadas en el método científico” ^10^. 

Se debe resaltar que la actual Ley Universitaria peruana 30220 ^12^, en su artículo 48, estipula que “la investigación constituye una función esencial y obligatoria de la universidad” y que “los docentes, estudiantes y graduados participan en la actividad investigadora”. Además, el artículo 79 de esta misma ley establece la investigación como función de los docentes universitarios, incluyendo dentro de sus deberes el “generar conocimiento e innovación a través de la investigación”. Por ello, el docente universitario está en la obligación de investigar y enseñar desde la experiencia, hecho que contribuirá a la formación de futuros investigadores, más aún en los años iniciales de la carrera. De allí el término “docente-investigador”.

### Producción científica del docente odontológico universitario peruano

El Perú ocupa el puesto 68 a nivel mundial en cuanto a investigación dentro de las profesiones de salud ^13^ y el puesto 56 en la investigación ligada con la Odontología ^14^. En este contexto, las universidades que aportan con mayor porcentaje a la producción científica del país son la Universidad Nacional Mayor de San Marcos y la Universidad Peruana Cayetano Heredia ^5^. Al considerar estas cifras, y teniendo en cuenta que la mayor producción científica universitaria proviene de la actividad investigativa docente, se podría afirmar que los docentes de ambas universidades son aquellos que más producen literatura científica dentro del ámbito de salud universitario en el Perú. Sin embargo, es importante considerar que el registro de producción científica se toma a partir de la filiación institucional colocada en el artículo publicado; en consecuencia, y sin olvidar los múltiples centros laborales en los que suele desenvolverse un docente universitario peruano (enseña en más de una universidad), las cifras que establecen dicho *ranking* podrían variar en mayor o menor medida si se tomara en cuenta la universidad de procedencia o “principal” en la que se desempeña el investigador. Existen preferencias por investigar en uno u otro centro en función de los incentivos brindados.

Un estudio realizado por Roman-González *et al.*^15^ determinó, al comparar la producción científica de las universidades peruanas, de acuerdo tanto con las publicaciones indizadas en Scopus como con el tamaño de las instituciones en cuestión, que la Pontificia Universidad Católica del Perú, la Universidad Peruana Cayetano Heredia y la Universidad Nacional Mayor de San Marcos ocupan, en ese orden, los tres primeros puestos dentro de la producción científica universitaria peruana. Se debe destacar que son el segundo y tercer puesto los que priman en la investigación relacionada a las ciencias de la salud. Estos resultados coinciden en gran medida con los reportados por Huaraca-Hilario *et al*. ^5^, aunque el orden de las dos universidades más destacadas no es el mismo debido a los diferentes criterios de evaluación considerados.

Al notar que la producción científica de docentes peruanos reportada en la literatura suele clasificarse como baja, surgen interrogantes respecto de los motivos: ¿cuáles son las principales trabas para la producción científica docente? Un estudio llevado a cabo por Barrutia *et al*. ^16^ tuvo entre sus objetivos responder dicha pregunta, para lo cual comparó la producción científica llevada a cabo por docentes en dos universidades peruanas, y concluyó que la principal causa por la que se reduce el tiempo dedicado a estos fines es la poca o nula remuneración que brindan las instituciones por realizar trabajos investigativos (46% de docentes entrevistados dio esta respuesta). Otras trabas mencionadas en las entrevistas fueron la falta de asesoría (21%) y de capacitación técnica (15%).

Teniendo en cuenta este reporte, y que la baja producción científica se debe principalmente a la percepción de un inadecuado sistema de remuneración para los investigadores universitarios, no es de extrañar que los docentes busquen la mejor opción remunerativa al establecer su filiación institucional o, en otros casos, resten importancia y tiempo para la actividad investigativa. Estas y otras trabas para la producción científica docente deben ser atendidas para revertir la situación.

### Diferencia entre investigación científica e investigación formativa

La investigación científica puede describirse como una práctica investigativa que cuenta con las particulares características de ser exigente y rigurosa tanto con los resultados que se obtienen como con el investigador que lleva a cabo el estudio. Está constituida por un conjunto de procedimientos teóricos, metodológicos y técnicos que tienen como finalidad obtener la respuesta a un problema planteado y cuya solución se encuentra en búsqueda ^17^; en cambio, la investigación formativa refiere al proceso de enseñanza-aprendizaje en el que el sujeto -estudiante universitario, en este caso- es expuesto a la literatura científica ya publicada y, mediante su análisis crítico, aprende a evaluarla y a sumarla en sus conocimientos ^18^, mientras se familiariza con los aspectos de la metodología investigativa. Además, la investigación formativa puede también ser descrita como la enseñanza a través de la investigación, pero se deben tener en cuenta dos características: la investigación es dirigida y guiada por un docente investigador, y el proceso es realizado por un sujeto en formación ^19^.

En ese contexto, la investigación formativa debe entenderse como un proceso en el que docentes y estudiantes trabajan juntos con el objetivo de inculcar en estos últimos las habilidades necesarias para introducirse en el ámbito investigativo: correcta redacción científica y excelente búsqueda bibliográfica, como pilares fundamentales ^20^; análisis estadístico y otros que van a ser de gran utilidad al momento de generar conocimientos mediante el método científico. Así, el estudiante contará con los conocimientos y las herramientas que le permitirán incursionar en la investigación científica.

### Importancia de la investigación formativa en el pregrado odontológico

Dentro de las ciencias de la salud, la renovación del conocimiento es constante; las nuevas evidencias que se generan permiten actualizar protocolos de atención e incorporar técnicas, materiales, instrumental y otros. Ya que la investigación formativa busca instaurar la cultura investigativa en los estudiantes en formación -pregrado odontológico, en nuestro contexto-, esta se puede considerar como el primer paso hacia la generación de nuevo conocimiento o como la aproximación inicial de futuros investigadores hacia un campo de posible interés. Un adecuado proceso de investigación formativa en el pregrado busca que el estudiante se acerque de forma gradual y sostenible al proceso investigativo, familiarizándose con actividades que en un futuro deberían ser parte de su quehacer diario, y que le permitan prepararse para lograr investigaciones bien ejecutadas y con resultados de calidad ^21^, que contribuyan a la continua renovación del conocimiento que caracteriza el campo de la salud. 

La difusión de la producción intelectual también es parte de la investigación formativa, pues la publicación es el paso final del proceso investigativo. En el contexto del pregrado, esto contribuye a que el estudiante fortalezca su pertenencia a la institución en cuestión (universidad) y, a la par, inicie su posicionamiento dentro del campo investigativo. Si bien la publicación se plantea como objetivo de la investigación, al hablar de investigación formativa se enfatiza y prioriza el aprendizaje que pueda obtenerse durante el proceso de investigar. No obstante, a medida que el estudiante se encuentra más próximo a terminar el pregrado, el enfoque puede tornarse hacia la buena realización del procedimiento; esto se dará en mayor o menor medida en función del nivel de aprendizaje en el que se encuentre el estudiante ^22^.

### Investigación formativa como base para la investigación científica

Cuando el estudiante está iniciando el curso de investigación, los docentes suelen hacer una inducción y asignación de tareas para que este consigne su proyecto. Es allí donde empieza el camino para el estudiante, un camino de selección y delimitación del problema que le interesa investigar ^23^. En ese contexto, es responsabilidad del docente universitario que los aprendizajes del estudiante sean significativos y él pueda asumir una postura crítica, tecnológica, académica y, sobre todo, humana respecto de lo que investiga y por qué lo está investigando.

Al asumir responsabilidad de un proyecto investigativo, el estudiante desarrolla un compromiso con el mismo y, sumado a la motivación docente, se ve impulsado a desarrollar las habilidades y adquirir los conocimientos que la investigación formativa le plantea ^24^. Hoy en día, el docente investigador es considerado un pilar fundamental en la formación de la nueva generación de estudiantes, es decir, la formación de personas agudas y críticas de pensamiento, que sean capaces de asumir cambios frente a la situación que les toca vivir y, a partir de ello, construir una mejor sociedad ^25^. Esta integración de habilidades y compromiso social adquiridos guían al estudiante hacia el desarrollo de investigación no solo durante su etapa de pregrado, sino también posteriormente; de esta manera, se plantea la transición de la investigación formativa hacia la científica.

Al hablar de la investigación formativa como base para la investigación científica, como hemos visto, se deben tener en cuenta factores cognitivos y motivacionales. Se plantea una línea en la que una buena experiencia en la formación del estudiante dentro de la investigación lleva a que se facilite su incursión en la aplicación rigurosa de lo aprendido para llegar al siguiente nivel: la investigación científica. Al llevar estos conceptos a la odontología, el profesional no solo debe poseer los conocimientos y la motivación, sino que debe estar familiarizado con la manera en la que se llevan a cabo las investigaciones, a fin de poder discernir qué evidencia científica es realmente válida y aplicable en su medio. Esto le permitirá planificar y ejecutar tratamientos adecuados para las situaciones clínicas que se le presenten ^26^ y, al adquirir la experiencia de aplicar la literatura, determinar qué vacíos considera que puede llenar mediante la investigación, con lo que establecerá una línea investigativa.

### Investigación formativa en las universidades peruanas

La investigación formativa se ha convertido en un tema de interés que abre un espacio de formación orientado a reflexionar, problematizar e indagar. Actualmente, la investigación en el Perú es un importante pilar de la estructura jerárquica universitaria. Como uno de los fines y funciones de la universidad, constituye una herramienta clave para formar profesionales íntegros, con competencias que la profesión y la sociedad necesitan. Sin embargo, pese a la orientación hacia el desarrollo de la investigación en general, la normatividad peruana no manifiesta del todo a la investigación formativa, como sí ocurre en otros países de Latinoamérica ^22^. 

Las universidades peruanas de reciente creación, especialmente aquellas particulares, y algunas de las primeras en ser fundadas, no evidencian información fidedigna acerca de la ejecución de su plan de desarrollo; incluso, no indican el seguimiento realizado a los estudiantes para que estos puedan adquirir las competencias necesarias, así como desarrollarse de manera exitosa hasta graduarse y obtener el título que les permita ejercer como profesionales ^26^. Por ello, es poco el seguimiento que se le da al desarrollo de las competencias investigativas.

Es cierto que existen casos como el de la Universidad Nacional de San Agustín de Arequipa (UNSA), donde se establece un proceso en el cual se induce a establecer precisiones respecto de la investigación científica y formativa, que se pueden vincular con el pensamiento crítico, la competitividad, el liderazgo, la libertad de pensamiento y de cátedra. Esta universidad se maneja mediante principios como la formación de espacios institucionales, inclusión en el currículo de metodologías que inducen a la libertad de pensamiento, creación y funcionamiento de incubadoras de proyectos, desarrollo de sistemas flexibles de enseñanza y programas de participación ^27^. No obstante, son pocas las universidades que siguen esta modalidad, y se observan más bien aplicaciones no reglamentadas o sin seguimiento estricto, lo que genera una aplicación parcial de la investigación formativa.

El Perú está encaminado hacia la investigación y no olvida su importancia. La finalidad investigativa de las universidades sigue intacta y el desarrollo de programas investigativos sigue en pie; sin embargo, como se ha comentado a lo largo del artículo, quedan vacíos por completar con el fin de aplicar de la mejor manera la formación en investigación, lo que se vería reflejado en una mayor y mejor investigación científica en el país.

## CONCLUSIONES

La producción científica docente peruana es baja, lo que se refleja en la también baja producción estudiantil, así como en el prestigio de los centros educativos a los cuales estos pertenecen. Se deben implementar políticas que permitan a los docentes superar las trabas manifestadas para que puedan cumplir con los objetivos de su labor docente-investigativa.

La investigación formativa es una estrategia de enseñanza aprendizaje que lleva a la planificación de las clases; por ende, implica pensar antes de hacer. Este camino de empezar a investigar es guiado por el docente, quien muchas veces logra ser motivador para que el alumno se interese en desarrollar más investigación. Esta estrategia, aplicada en universidades peruanas que imparten la carrera de odontología, demuestra un crecimiento en producción científica, pero que aún no es óptimo. 

## Financiamiento:

Autofinanciado
